# Development and validation of a clinical prediction model of fertilization failure during routine IVF cycles

**DOI:** 10.3389/fendo.2023.1331640

**Published:** 2024-01-19

**Authors:** Liu Xingnan, Zhang Na

**Affiliations:** Department of Reproductive Medicine, The Fourth Hospital of Hebei Medical University, Shijiazhuang, China

**Keywords:** *in vitro* fertilization, rescue ICSI, clinical prediction model, fertilization failure, nomogram

## Abstract

**Purpose:**

This study aims to create and validate a clinical model that predict the probability of fertilization failure in routine *in-vitro* fertilization (IVF) cycles.

**Methods:**

This study employed a retrospective methodology, gathering data from 1770 couples that used reproductive center’s of the Fourth Hospital of Hebei Medical University standard IVF fertilization between June 2015 and June 2023. 1062 were in the training set and 708 were in the validation set when it was randomly split into the training set and validation set in a 6:4 ratio. The study employed both univariate and multivariate logistic regression analysis to determine the factors those influence the failure of traditional *in vitro* fertilization. Based on the multiple regression model, a predictive model of traditional IVF fertilization failure was created. The calibration and decision curves were used to assess the effectiveness and therapeutic usefulness of this model.

**Results:**

The following factors independently predicted the probability of an unsuccessful fertilization: infertility years, basal oestrogen, the rate of mature oocytes, oligoasthenozoospermia, sperm concentration, sperm vitality, percentage of abnormal morphological sperm, and percentage of progressive motility (PR%).The receiver operating characteristic curve’s area under the curve (AUC) in the training set is 0.776 (95% CI: 0.740,0.812), while the validation set’s AUC is 0.756 (95% CI: 0.708,0.805), indicating a rather high clinical prediction capacity.

**Conclusion:**

Our generated nomogram has the ability to forecast the probability of fertilization failure in couples undergoing IVF, hence can assist clinical staff in making informed decisions.

## 1 Introduction

Fertilization failure, including total fertilization failure (TFF), is defined as a normal fertilization rate of less than 30% during *in vitro* fertilization-embryo transfer (IVF-ET) aided pregnancy (1). Failure to fertilize is inevitable and typically occurs in the range of 5% to 15%. The inability of couples with infertility issues to conceive is a contributing factor to fertilization failure, along with factors related to sperm and egg quality, the fertilization process, and the hormonal environment (2). Currently, it is suggested that oocyte activation failure (OAF) seems to be the primary cause of TFF (3). Sperm plays a vital role in fertilization, oocyte division, and paternal inheritance, making it a significant factor in fertilization failures (4). A significant number of fertilization failures are closely related to semen quality and low sperm function, including patients with severe, weak, and dysmospermia. Additionally, female factors such as oocytes maturity and oocytes quality are also associated with fertilization failure.

Rescue intracytoplasmic sperm injection (R-ICSI), also known as rescue ICSI, is widely used in reproductive centers to address IVF fertilization failure (5). Its goal is to improve the utilization rate of oocytes and reduce the psychological and economic burden on infertile couples caused by inadequate fertilization of precious oocytes. However, some studies suggested that rescue ICSI may result in lower later fertilization rates and clinical pregnancy rates due to the prolonged culture time of oocytes and slow embryo growth, which can leads to a mismatch with the endometrium (6). While ICSI can effectively address most cases of fertilization failure by directly injecting sperm into the oocyte plasma using micromanipulation, its use is limited to severe sperm factors and clear fertilization disorders. Moreover, the high cost and time-consuming nature of ICSI make it an unfavorable alternative to conventional IVF for pregnancy assistance (7). Therefor, accurately, predicting the probability of fertilization failure in advance is crucial to guide clinical workers in selecting the appropriate insemination methods and minimizing oocyte wastage. The objective of this paper is to develop a nomogram for predicting IVF fertilization failure.

## 2 Materials and methods

### 2.1 Patients’ selection

A retrospective study was conducted on couples undergoing IVF and Rescue ICSI cycles at the Fourth Hospital of Hebei Medical University from June 2015 to June 2023. According to the World Health Organization’s definition, infertility refers to couples who have been unable to conceive after at least one year unprotected intercourse (8). All participants in this study were infertile couples treated at our reproductive center. Infertility is categorized into primary infertility and secondary infertility. Primary infertility refers to the inability to conceive for more than one year, while secondary infertility refers to inability to conceive again for more than one year after a previous pregnancy (9). The inclusion criteria for the study were couples with a potential for fertilization failure. The following were the exclusion criteria: 1. Females with abnormal zona pellucida, such as indented zona pellucida; 2. Couples requiring sperm assistance due to the husband’s azoospermia; 3. Couples with chromosomal abnormalities or cancer; 4. Couples who did not undergo insemination or have oocytes retireved during the cycle. Informed consent was obtained from all participants. All infertile couples included in the study signed informed consent prior to undergoing IVF. A total of 1770 couples were included in the study, with 264 of them using rescue-ICSI due to fertilization failure.

### 2.2 The procedure of IVF

#### 2.2.1 Controlled ovulation stimulation protocol

The individualized ovarian stimulation program is designed based on the female’s age, basic hormone levels, and ovarian reserve function. This program involves monitoring follicle growth, through transvaginal ultrasound, and adjustments are made according to the size of the dominant follicle and hormone levels. When the appropriate criteria are met, the patient is injected with human chorionic gonadotropin (hCG) or gonadotropin releasing hormone (GnRH-a), followed by a vaginal vault puncture to retrieve the oocytes. This procedure is conducted 36 hours after the trigger day.

#### 2.2.2 Semen treatment and *in vitro* fertilization

On the day of oocyte retrieval, the male partner provided sperm by masturbating. After that, the sperm underwent density gradient centrifugation. Afterwards, the oocytes and sperm were incubated together for 4 hours. Then, the granulosa cells surrounding the oocytes were removed to make it easier to observe the discharge of the second polar body and the fertilization process (10). Fertilization failure is defined as a discharge rate of the second polar body of less than 30%, or when no discharge of the second polar body is apparent. In such cases, rescue-ICSI intervention becomes necessary.

### 2.3 Data collection

Age, Body Mass Index (BMI), occupation, type of infertility, years of infertility, and clinical diagnosis were recorded as the clinical parameters for both infertile couples. The levels of basal estrogen, luteinizing hormone (LH), and follicle stimulating hormone (FSH) in females, as well as estrogen, progesterone, and LH levels on the trigger day, were also measured. The mature follicular rate was assessed, along with semen parameters on the day of oocyte retrieval, including semen volume, percentage of morphologicaliy abnormal sperm, sperm vitality, sperm concentration, and PR%. A fertilization rate of less than 30%, or the expulsion of the second polar body of less than 30%, referred to as an IVF fertilization failure, was considered a positive event.

### 2.4 Statistical analysis

According to the random sampling technology, the infertile couples were divided into training set and validation set in a 6:4 ratio. The continuous variables were represented as the mean ± standard deviation (SD), while non-normally distributed data were presented as the median (interquartile range). To compare variables between groups, Student’s t-tests (for normally distributed data) or the Mann–Whitney U-test (for non-normally distributed data) were employed. Categorical variables were expressed as percentages, and the chi-squared test was used for statistical comparison. These data were analyzed using SPSS 23.0.

Univariate logistic regression was used to identify predictive factors associated with fertilization failure. The variables with *P*<0.05 were entered into the next multifactor analysis. To reduce overfit bias, internal validation was performed using bootstrap resampling. Bootstrapping repeated the process of drawing samples with replacement from the original dataset 500 times. The closer the original and corrected statistics, the better the fit of the regression model (11). The area under the ROC curve was used to assess the accuracy of the nomogram. Additionally, a decision curve analysis was performed to determine the clinical utility of this model. The statistical analysis mentioned above was conducted using IBM SPSS Statistics for Windows (version 23.0) and R (version 4.3.1). *P* value less than 0.05 was considered statistically significant.

## 3 Results

### 3.1 Baseline characteristics

A total of 1770 groups of infertile couples were included in the study and analyzed after applying the inclusion and exclusion criteria. Out of these, 264 couples (14.9%) experienced failed fertilization, which was defined as a fertilization rate of less than 30%. The infertile couples were divided into a training set (n=1062) and a validation set (n=708) in a 6:4 ratio for constructing and testing the model. The basic characteristics, summarized in **Table 1**, showed no statistically significant difference in baseline characteristics between the two groups (*P*<0.05).

**Table 1:** Basic characteristics of study participants in the training and validation sets.

Characteristics	Validation set (n=708)	Training set (n=1062)	P-Value
Female age	30.0 [28.0;34.0]	30.0 [27.0;33.0]	0.582
Female profession			0.100
Ordinary occupation	701 (99.0%)	1059 (99.7%)	
High-risk occupation	7 (0.99%)	3 (0.28%)	
Male age	30.0 [28.0;34.0]	31.0 [28.0;34.0]	0.786
Male profession			1.000
Ordinary occupation	682 (96.3%)	1024 (96.4%)	
High-risk occupation	26 (3.67%)	38 (3.58%)	
Female Infertility type			0.147
primary infertility	519 (73.3%)	812 (76.5%)	
secondary infertility	189 (26.7%)	250 (23.5%)	
Infertility years	3.00 [2.00;5.00]	3.00 [2.00;5.00]	0.569
Female BMI (Kg/m^2^)	23.6 [21.2;27.2]	23.4 [20.8;26.6]	0.116
PCOS			0.991
No	547 (77.3%)	819 (77.1%)	
Yes	161 (22.7%)	243 (22.9%)	
Endometriosis adenomyosis			0.720
No	634 (89.5%)	944 (88.9%)	
Yes	74 (10.5%)	118 (11.1%)	
Pelvic inflammation and tubal disease			0.538
No	406 (57.3%)	592 (55.7%)	
Yes	302 (42.7%)	470 (44.3%)	
Thyroid disease			0.300
No	688 (97.2%)	1021 (96.1%)	
Yes	20 (2.82%)	41 (3.86%)	
Male Infertility type			0.908
primary infertility	583 (82.3%)	878 (82.7%)	
secondary infertility	125 (17.7%)	184 (17.3%)	
Male BMI (Kg/m^2^)	25.4 [22.9;27.8]	25.9 [23.1;28.4]	0.027
Teratozoospermia			0.508
No	282 (39.8%)	441 (41.5%)	
Yes	426 (60.2%)	621 (58.5%)	
Oligoasthenozoospermia			0.371
No	515 (72.7%)	794 (74.8%)	
Yes	193 (27.3%)	268 (25.2%)	
Starting dosage of Gn used (IU)	200 [150;225]	200 [150;225]	0.523
Characteristics	Validation set (n=708)	Training set (n=1062)	P-Value
Total dosage of Gn used (IU)	2488 [1875;3300]	2475 [1875;3375]	0.971
The duration of treatment of Gn	12 [10;15]	14 [12;17]	0.421
E2 on the day of HCG trigger (pg/ml)	2526 [1493;3240]	2598 [1582;3448]	0.338
LH.on.the.day.of.HCG.trigger (IU/L)	1.06 [0.69;1.98]	1.14 [0.69;2.15]	0.072
P on the day of HCG trigger (ng/ml)	0.73 [0.47;1.12]	0.78 [0.50;1.16]	0.113
The number of oocytes retrieved	12.0 [8.00;16.0]	11.0 [7.00;15.0]	0.311
the rate of mature oocytes (%)	0.91 [0.78;1.00]	0.91 [0.80;1.00]	0.195
Semen volume (ml)	2.50 [2.00;3.00]	2.50 [2.00;3.00]	0.373
sperm vitality (%)	40.0 [35.0;45.0]	40.0 [35.0;45.0]	0.311
sperm concentration (10^6^/ml)	40.0 [30.0;50.0]	40.0 [30.0;55.0]	0.659
PR%	35.0 [30.0;40.0]	35.0 [30.0;40.0]	0.185
percentage of abnormal morphological sperm (%)	97.0 [96.0;98.0]	97.0 [96.0;98.0]	0.494
Basal estrogen (pg/ml)	37.6 [27.6;52.1]	37.2 [26.0;50.3]	0.350
Basal LH (IU/L)	4.70 [3.12;6.63]	4.57 [3.12;6.45]	0.458
Basal FSH (IU/L)	6.18 [5.11;7.46]	6.29 [5.07;7.49]	0.547

Continuous variables are shown as the median (interquartile range) or mean ± standard deviation. Categorical variables are presented as percent.Student’s t-tests (for normally distributed data) or the Mann–Whitney U-test (for non-normally distributed data) were employed. Categorical variables were expressed as percentages, and the chi-squared test was used for statistical comparison.

BMI, body mass index; FSH, follicle-stimulating hormone; LH, luteinizing hormone; P, progesterone; E2, estradiol; High-risk occupation: Occupations with high temperature, toxic gas, or chemical exposure. Training set vs. validation set: P < 0.05.

### 3.2 Logistic regression analysis

**Table 2** displays the results of the univariate logistic regression analysis for fertilization failure. It reveals that in the failed fertilization group, the percentage of abnormal morphological sperm, P on the day of HCG trigger, oligoasthenozoospermia, teratozoospermia and the number of infertility years for couple significantly increased. On the other hand, the basal estrogen in females, PR%, sperm vitality, sperm concentration, number of oocytes retrieved, and the rate of mature oocytes significantly reduced the likelihood of fertilization failure. Variables with a *P* value <0.05 were considered statistically significant and included in the subsequent multivariate analysis.

**Table 2:** Univariate analysis in the training set.

Variables	OR	CI	*P*-Value
Basal estrogen (pg/ml)	0.993	(0.986-0.998)	0.025
Percentage of abnormal morphological sperm (%)	1.415	(1.23-1.634)	<0.001
PR%	0.953	(0.932-0.974)	<0.001
Sperm vitality (%)	0.971	(0.951-0.991)	0.005
Sperm concentration (10^6^/ml)	0.979	(0.969-0.988)	<0.001
The rate of mature oocytes (%)	0.094	(0.045-0.196)	<0.001
Number of oocytes retrieved	0.94	(0.911-0.969)	<0.001
P on the day of HCG trigger (ng/ml)	1.19	(1.012-1.423)	0.04
Oligoasthenozoospermia	2.113	(1.479-3.002)	<0.001
Teratozoospermia	1.76	(1.234-2.543)	0.002
Infertility years	1.082	(1.024-1.14)	0.004

OR, odds ratio; CI, confidence interval.

The results of the multiple logistic regression analysis presented in **Table 3**. According to **Table 3**, the independent predictors of fertilization failure were infertility years (OR: 1.063, 95%CI: 1.002,1.126, *P*=0.038), the rate of mature oocytes (OR: 0.106, 95%CI:0.046,0.239, *P*<0.001), number of oocytes retrieved (OR: 0.939, 95%CI:0.906,0.971, *P*<0.001), basal estrogen level (OR: 0.991, 95%CI:0.984,0.998, *P*=0.015), oligoasthenozoospermia (OR: 1.512, 95%CI:1.013,2.241, *P*=0.041), sperm concentration (OR: 0.982, 95%CI: 0.971,0.993, *P*=0.001), sperm vitality (OR: 1.177, 95%CI: 1.086,1.282, *P*<0.001), percentage of abnormal morphological sperm (OR: 1.312, 95%CI: 1.122,1.543, *P*=0.001) and PR% value of male semen (OR: 0.821, 95%CI: 0.751,0.894, *P*<0.001).

**Table 3:** Multivariate logistic regression model in the training set.

Variables	OR	CI	P-Value
Infertility years	1.063	(1.002-1.126)	0.038
Oligoasthenozoospermia	1.512	(1.013-2.241)	0.041
Sperm concentration (10^6^/ml)	0.982	(0.971-0.993)	0.001
Sperm vitality (%)	1.177	(1.086-1.282)	<0.001
PR%	0.821	(0.751-0.894)	<0.001
Percentage of abnormal morphological sperm (%)	1.312	(1.122-1.543)	0.001
Basal estrogen (pg/ml)	0.991	(0.984-0.998)	0.015
Number of oocytes retrieved	0.939	(0.906-0.971)	<0.001
The rate of mature oocytes (%)	0.106	(0.046-0.239)	<0.001

### 3.3 Development and Validation of the clinical prediction model

**Figure 1:**
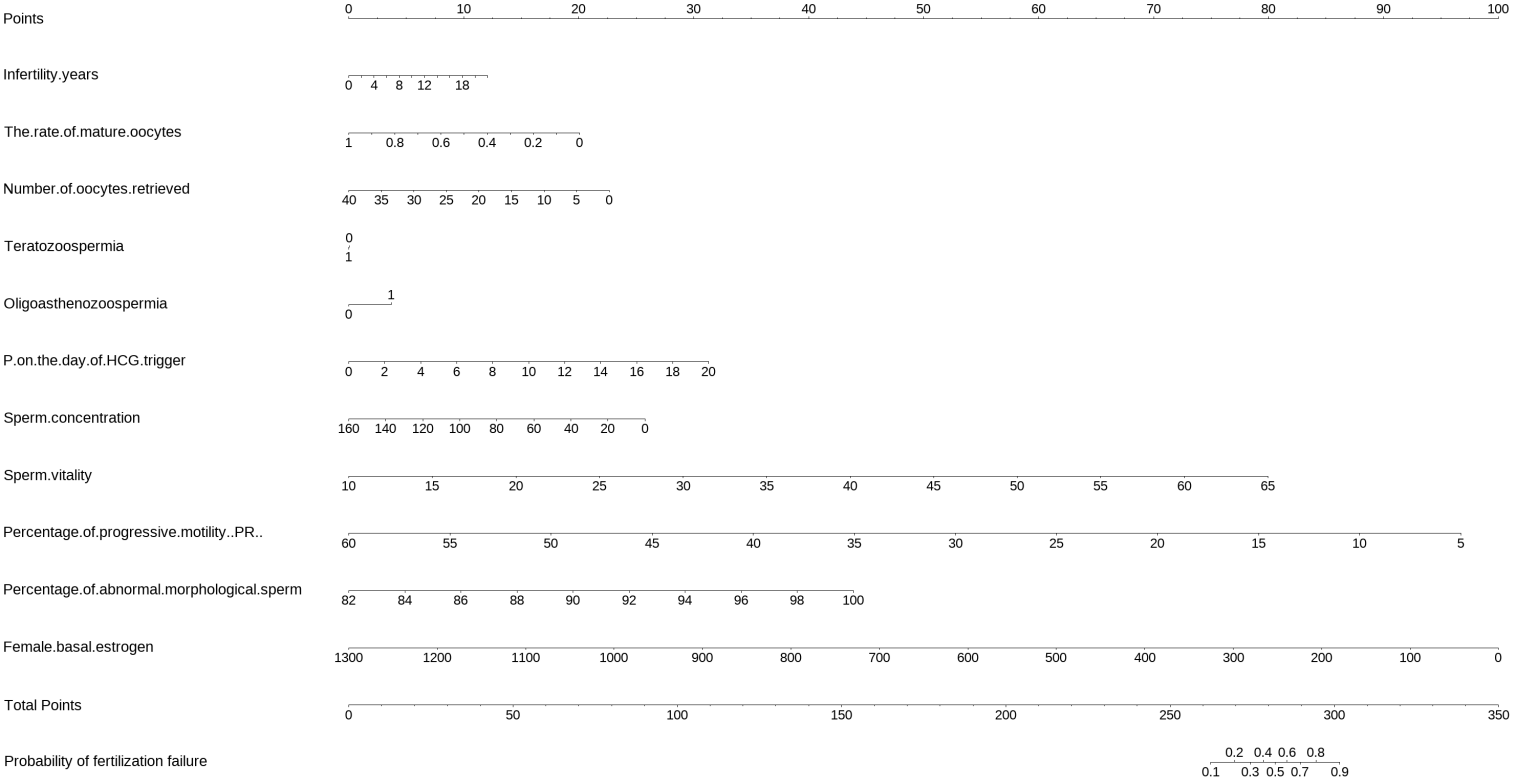
The nomogram to predict the probability of fertilization failure occurring in routine *in-vitro* fertilization (IVF) cycles. The nomogram can be applied by following procedures: draw a line perpendicular from the corresponding axis of each risk factor until it reaches the top line labeled “Points”; sum up the points for all risk factors and recorded as the total score; and draw a line descending from the axis labeled “Total points” until it intercepts the lower line to determine the probability of failed fertilization. The optimal threshold point was calculated using receiver operating characteristic (ROC) curve.

**Figure 2:**
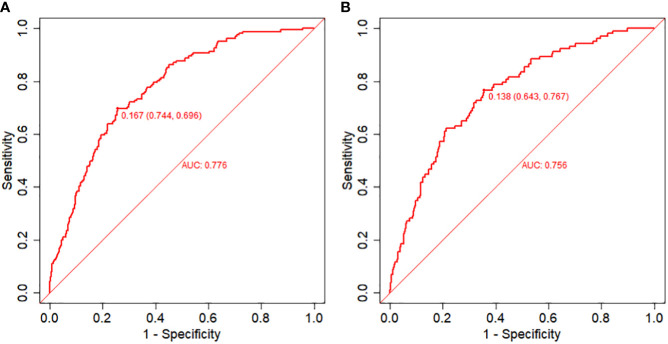
Receiver operating characteristic (ROC) curves and calibration plots of the training and validation sets. **(A)** Area under the ROC curve (AUC) of the training set is 0.776 (95% CI: 0.740,0.812). **(B)** AUC of the validation set is 0.756 (95% CI: 0.708,0.805). .

**Figure 3:**
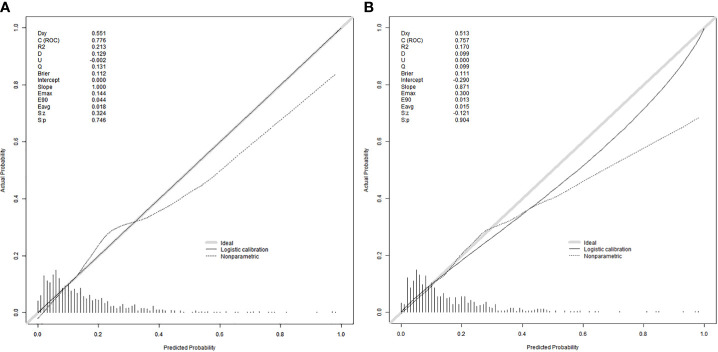
Calibration curves were used to evaluate the calibration of the model. The horizontal axis is the predicted probability provided by this model, and the vertical axis is the observed incidence of pregnancy failure. The ideal line with 45° slope represents a perfect prediction (the predicted probability equals the observed probability). The lower the Brier score for a set of predictions, the better the prediction calibration. When the slope was closer to 1.00, the prediction model had better calibration power. **(A)** Calibration curve for training set (Brier = 0.112, Slope = 1.000). **(B)** Calibration curve for validation set (Brier = 0.111, Slope = 0.871).

**Figure 4:**
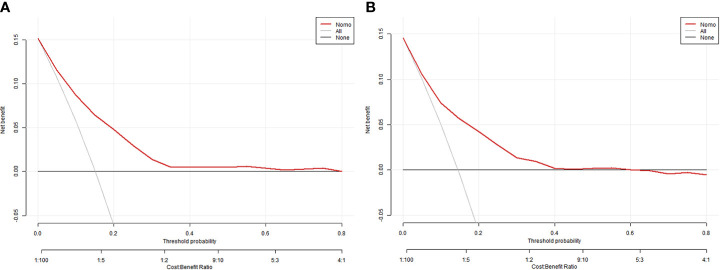
the decision curve analysis of the training set **(A)** and validation set **(B)**.

The equations were constructed using regression coefficients to determine the probability of fertilization failure (P) = -24.78 + 0.061* Infertility years - 0.008 *level of female basic estrogen -2.25 *rate of mature oocytes -0.63* number of occytes retrieved- 0.018 * sperm concentration + 0.163 * sperm vitality - 0.197 * percentage of progressive motility (PR%) + 0.27 * percentage of abnormal morphological sperm + 0.413 * Oligoasthenozoospermia. To predict the probability of fertilization failure in conventional IVF assisted couples, we developed a nomogram (**Figure 1**), that includes of the above independent predictors. The area under the receiver operating characteristic curve (AUC) for the training set (**Figure 2A**) is 0.776 (95% CI: 0.740,0.812), indicating good clinical predictive ability. Similarly, the validation set (**Figure 2B**) has an AUC of 0.756 (95% CI: 0.708,0.805). The calibration curves for the training set (**Figure 3A**) and validation set (**Figure 3B**) have slopes of 1.000 and 0.891, respectively, indicating good calibration ability. Furthermore, the decision curve analysis of the training set (**Figure 4A**) and validation set (**Figure 4B**) demonstrates that the prediction model has high net income and clinical application value, as it is positioned higher on the decision curve.

## 4 Discussion

### 4.1 Multivariate analysis affecting IVF fertilization failure

With the advancement of assisted reproductive technology, the successful implementation of IVF technology in clinical practice has brought hope to couples struggling with infertility. However, the success rate of *in vitro* fertilization is still not promising. Research has indicated that factors such as age (12), sperm quality (13) and oocyte quality play a crucial role in determining the success rate of IVF-ET.

This study is a retrospective analysis based on the assisted reproductive population. Infertile couples who underwent by conventional IVF and rescue ICSI were selected from the Department of Reproductive Medicine at the Fourth Hospital of Hebei Medical University, between June 2015 and June 2023. The objective of this study was to compare the basic information and semen parameters of husbands in failed and successful couples. The results revealed that several factors, including the percentage of progressive motility, sperm concentration, sperm vitality, percentage of abnormal morphological sperm, oligoasthenozoospermia, basal estrogen level in females, rate of mature oocytes, and duration of infertility, were identified as independent predictors of fertilization failure.

It has been suggested that fertilization failure may be attributed to disruptions in the interaction between sperm and oocytes at the level of the cumulus cell and oocyte zona pellucida (14). In this study, it is believed that the rate of sperm motility plays a crucial role in the success of fertilization, as there are varying rates of sperm survival and significant differences in the fertilization rate (15). Our study demonstrates that males with oligoasthenospermia or decreased sperm density, motility, and PR% have a significantly lower fertilization rate, which is consistent with previous findings (16, 17). Additionally, our study found that female factors also influence the likelihood of fertilization failure. Through the analysis in **Table 3**, it was observed that an increase in female baseline estrogen levels and the rate of mature oocytes decreased the probability of fertilization failure, aligning with previous research (18). We hypothesize that estrogen levels impact oocyte quality, and mature oocytes contribute to the successful binding of sperm and oocytes. Several studies have emphasized differences in the number of follicles, the total number of obtained oocytes, and the number of mature oocytes in the fertilization failure group (19). This could be linked to mechanisms such as meiotic spindle abnormalities in oocytes (20). Furthermore, this study revealed a significant difference in the number of years of infertility between the two groups, with a statistically significant distinction.

In the occurrence factors of infertility in China, 20-30% are caused by both couples. Semen parameters are important indicators used to evaluate male fertility and commonly employed in clinical practice. According to the WHO guidelines, important parameters for evaluating semen quality include the percentage of progressive motility, sperm concentration, sperm vitality, and the percentage of abnormal morphological sperm. The predictive value of these semen analysis parameters in forecasting the outcome of IVF fertilization has been established (21). Approximately 20% of IVF treatment cases are associated with a low fertilization rate or complete fertilization failure. Studies have indicated that fertilization failure is often linked to poor semen quality, specifically oligoasthenospermia, hypomospermia, and low sperm and oocyte union. However, the exact cause is still unclear (22, 23).

While sperm factors are the primary cause of fertilization disorders, oocyte abnormalities also contribute to the issue. Research has shown a correlation between the maturity and quality of the oocytes and the success rate of fertilization (24).

### 4.2 Development and validation of the nomogram prediction model

The successful combination of sperm and eggs marks the beginning of embryo formation and is a crucial step in *in vitro* fertilization embryo transfer. To assist clinical IVF in selecting appropriate insemination methods and guiding clinical work, we developed prediction models based on retrospective clinical studies, which include independent predictive factors that affect fertilization failure. In the field of reproductive medicine, there have been numerous previous studies on clinical prediction models of assisted reproductive technology (ART) for pregnancy, and most models have shown an area under the curve ranging between 0.59 and 0.8 (25). The area under the curve (AUC) of the modeling set in this study is 0.776, indicating a moderate predictive effect. An article published in JAMA in 2022 emphasizes calibration as an important indicator of predictive models, as it reflects their ability to accurately estimate absolute risk. The article also suggests the inclusion of a calibration curve in clinical papers on predictive models (26). For this study, we randomly allocated 60% of the samples to the modeling group and 40% to the validation group, and the model performed well in both groups. In recent years, the clinical application value of prediction models has been emphasized, with the evaluation mainly based on decision curve analysis, which assesses whether the model can benefit patients by influencing clinical decisions (27). We generated decision curves for both groups and demonstrated that the nomogram model yielded a substantial net benefit.

The occurrence of fertilization failure in routine IVF cycles is influenced by factors such as semen quality, ovarian function, egg quality, and maturity. However, the impact of these factors is still being investigated. Considering this limitation, our objective was to develop a clinical model using retrospective data to predict fertilization failure. This model aims to assist in selecting appropriate insemination methods, thus avoiding the wastage of eggs. Furthermore, there is potential to incorporate additional variables in future iterations of the model. Given the relatively low rate of fertilization failure in regular IVF cycles (approximately 5%-15%), we analyzed the final nomogram, which demonstrated a maximum net gain of approximately 15% according to the decision curve.

Furthermore, the clinical selection of appropriate insemination methods for infertile couples is a topic under active investigation. Our model uses internal verification of the results of the nomogram, without external verification, which is a significant limitation of this paper. We hope to address this limitation by continuing the multicenter retrospective study to improve the generalizability of the results. Nonetheless, our exploration offers valuable insights for future research. We aim to conduct more comprehensive and in-depth studies to develop a systematic and comprehensive clinical prediction model.

## 5 Conclusions

We discovered that factors such as female infertility duration, basal estrogen levels, rate of mature oocytes, number of oocytes retrieved, oligoasthenozoospermia, sperm concentration, sperm vitality, percentage of abnormal morphological sperm, and PR% independently predicted the likelihood of fertilization failure. Our retrospective study has developed a well-calibrated model that accurately predicts the probability of fertilization failure in infertile couples undergoing routine IVF treatment. This model carries significant clinical implications.

## Data availability statement

The original contributions presented in the study are included in the article/**Supplementary Material**. Further inquiries can be directed to the corresponding author.

## Ethics statement

The studies involving humans were approved by Ethics Committee of Reproductive Medicine Department, The Fourth Hospital, Hebei Medical University. The studies were conducted in accordance with the local legislation and institutional requirements. The participants provided their written informed consent to participate in this study.

## Author contributions

ZN: Writing – review & editing. LX: Writing – original draft.
