# Supplementary material for: Development and validation of a clinical prediction model of fertilization failure during routine IVF cycles
Source: Front Endocrinol (Lausanne). 2024 Jan 19;14:1331640. doi: 10.3389/fendo.2023.1331640 (PMC10834765; doi:10.3389/fendo.2023.1331640)
Supplement: Supplementary file 1 [file DataSheet_1.docx]

**Data reduction：**

data==na.omit(data)

head(data)

**Set the factor type:**

data$sex<-as.factor(data$sex)

data$diagnosis<-as.factor(data$diagnosis)

data$T1<-as.factor(data$T1)

data$T2<-as.factor(data$T2)

**Production of the baseline table:**

data <- as.data.frame(data)

tab1 <-twogrps(data, gvar = "diagnosis")

write.csv(tab1$Table,file="tab1.csv")

**Collinearity analysis of data：**

numdata = data%>%

mutate(sex = as.factor(sex),

diagnosis = as.factor(diagnosis),

T1 = as.factor(T1),

T2 = as.factor(T2),)%>%

select(where(is.numeric))

**logistic**

**Dataset division:**

set.seed(1)

train_id = sample(1:nrow(data),0.85*nrow(data))

train=data[train_id,]

test=data[-train_id,]

write.csv(train,file="train.csv")

write.csv(test,file="test.csv")

**Hierogram production:**

mydata<-train

attach(mydata)

dd<-datadist(mydata)

options(datadist='dd')

fit0<-lrm(diagnosis ~ T1+T2+WBC+RBC+Lymph.+Mono.+RDW+A+AST+BUN+TG+PT,

data = mydata, x = T, y = T)

**fit0**

nom0 <- nomogram(fit0, fun = plogis,fun.at = c(.001,.01,.05,.5, .95, .99,.999),

lp = T, funlabel = "diagnosis rate")

plot(nom0)

fit1<-lrm(diagnosis ~ T1+T2+RBC+Mono.+RDW +AST+BUN,

data = mydata, x = T, y = T)

**fit1**

summary(fit1)

nomogram

nom1 <- nomogram(fit1, fun = plogis,fun.at = c(.001,.01,.05,.5, .95, .99,.999),

lp = T, funlabel = "diagnosis rate")

plot(nom1)

**Make the area curve under the curve:**

gd<-predict(fit1, newdata = train,

se.fit = FALSE, dispersion = NULL, terms = NULL,

na.action = na.pass)

gd2<-predict(fit1, newdata = test,

se.fit = FALSE, dispersion = NULL, terms = NULL,

na.action = na.pass)

**ROC**

library(pROC)

library(ggplot2)

**ROC of training set**

roc.list <- roc(train$diagnosis, gd)

roc.list

g.list <- ggroc(roc.list, alpha = 1 ,size = 0.8,legacy.axes = TRUE,color="red")

g.list+theme_classic2() + ggtitle("train")+annotate(geom = "segment", x = 0, y = 0, xend =1, yend = 1)

**ROC of testing set**

roc.list <- roc(test$diagnosis, gd2)

roc.list

g.list <- ggroc(roc.list, alpha = 1 ,size = 0.8,legacy.axes = TRUE,color="skyblue")

g.list <- ggroc(roc.list, alpha = 1 ,size = 0.8,legacy.axes = TRUE,color="red")

g.list+theme_classic2() + ggtitle("test")+annotate(geom = "segment", x = 0, y = 0, xend =1, yend = 1)

**Calibration curve making:**

ca11 <- calibrate(fit1, cmethod="hare",method="boot", B=1000,

xlab = "Nomogram Predicted Survival", ylab = "Actual Survival")

plot(ca11,xlim=c(0,1.0),ylim=c(0,1.0),

xlab = "Nomogram Predicted Survival", ylab = "Actual Survival")

**Decision curve making：**

library(rmda)

t1 <- train

t2 <- test

**Change the outcome to a numerical type variable：**

t1$diagnosis <- as.numeric(t1$diagnosis)

t1$diagnosis[t1$diagnosis == 2] <- 0

t2$diagnosis <- as.numeric(t2$diagnosis)

t2$diagnosis[t2$diagnosis == 2] <- 0

complex<-decision_curve(diagnosis ~ sdf+en+ALB+G,

data = t1,family = binomial(link ='logit'),

thresholds = seq(0,1, by = 0.01),

confidence.intervals= 0.95,

study.design = 'case-control',

population.prevalence= 0.3)

complex1<-decision_curve(diagnosis ~ II +endotracheal_intubation+ALB+G,

data = t2,family = binomial(link ='logit'),

thresholds = seq(0,1, by = 0.01),

confidence.intervals= 0.95,

study.design = 'case-control',

population.prevalence= 0.3)

List<- list(complex1,complex)

plot_decision_curve(List,

curve.names=c('test','train'),

cost.benefit.axis =FALSE,col= c('red','blue'),

confidence.intervals=FALSE,

standardize = FALSE)
